# Suppression of CCT3 inhibits the proliferation and migration in breast cancer cells

**DOI:** 10.1186/s12935-020-01314-8

**Published:** 2020-06-05

**Authors:** Gang Xu, Shanshan Bu, Xiushen Wang, He Zhang, Hong Ge

**Affiliations:** 1 Department of Radiation Oncology, Affiliated Cancer Hospital of Zhengzhou University, No. 127 Dongming Road, Zhengzhou, 450008 Henan China; 2 Department of Pathology, Affiliated Cancer Hospital of Zhengzhou University, No.127 Dongming Road, Zhengzhou, 450008 Henan China

**Keywords:** CCT3, Breast cancer, Proliferation, Metastasis, Cell cycle, Apoptosis

## Abstract

**Background:**

CCT3 is a subunit of chaperonin-containing TCP-1 (CCT), which folds many proteins involved in cancer development and plays an important role in many cancers. However, the role of CCT3 in breast cancer is still unclear.

**Methods:**

CCT3 expression was knocked down by transfecting breast cancer cells with lentiviral shRNA. The proliferation of breast cancer cells (HCC1937 and MDA-MB-231) was detected by Celigo image cytometry and MTT assay, the migration of the cells was measured by Transwell analysis, cell cycle distribution and apoptosis was detected by flow cytometry, and changes in signal transduction proteins were detected by western blot analysis.

**Results:**

The expression of CCT3 was significantly suppressed by transduction with lentiviral shRNA; CCT3 knockdown significantly reduced the proliferation and metastasis ability of breast cancer cells (HCC 1937 and MDA-MB-231), increased the proportion of cells in S phase, and decreased the proportion of cells in G1 phase compared to those in shControl cells. There was no significant change in the number of cells in the G2/M phase. Apoptosis analysis showed that knockdown of CCT3 induced apoptosis in breast cancer cells. Western blot analysis showed that the expression of many signal transduction proteins was changed after suppression of CCT3. A rescue experiment showed that overexpression of NFκB-p65 rescued the cell proliferation and migration affected by CCT3 in breast cancer cells.

**Conclusion:**

CCT3 is closely related to the proliferation and migration of breast cancer and may be a novel therapeutic target.

## Background

Breast cancer is a common malignant tumour in women. At present, the incidence rate of breast cancer is 24.2% worldwide. The mortality rate is also the highest among malignant tumours, accounting for approximately 15% of cancer-related deaths in women [1]. At present, the treatment of breast cancer mainly includes neoadjuvant therapy, surgery, chemotherapy, radiotherapy, targeted therapy and endocrine therapy [2]. The application of a comprehensive treatment mode improves the prognosis of breast cancer and prolongs the survival time of patients, but the overall effect is still unsatisfactory, especially for patients with stage IV metastasis, for whom the median total survival time is only 2–3 years [3]. Therefore, identification of a novel therapeutic target to treat breast cancer is an urgent need.

Chaperonins are molecules that assist in the folding of newly synthesized and stress-denatured polypeptide chains and are divided into two groups, group I and group II. Heat shock protein 60 (HSP60) or GroEL in bacteria belongs to group I, and chaperonin-containing TCP-1 (CCT or TRiC) belongs to group II. CCT is a large complex composed of two stacked rings, back-to-back, consisting of eight distinct subunits (CCT1-CCT8) [4–6]. In cancer cells, CCT folds proteins related to carcinogenesis, such as kirsten rat sarcoma viral oncogene (KRAS), Signal transducers and activators of transcription 3 (STAT3), and p53. CCT3 is an important subunit of CCT and is widely studied in different cancers. The mRNA and protein expression of CCT3 in hepatocellular carcinoma (HCC) tissues are higher than those in non-HCC tissues, and CCT3 plays an important role in the tumorigenesis and progression of HCC and has prognostic value in HCC [7, 8]. Further study showed that CCT3 is a novel regulator of spindle integrity and is required for proper kinetochore-microtubule attachment during mitosis [9]. In gastric cancer, a higher level of CCT3 expression was detected in tumour tissues than in non-cancerous epithelial tissues. Knockdown of CCT3 inhibited the proliferation and survival of gastric cancer cells, and gene expression analysis showed that CCT3 knockdown was associated with down-regulation of mitogen-activated protein kinase 7, cell division cycle 42(cdc42), cyclin D3 and up-regulation of cyclin-dependent kinase 2 and 6 [10]. In papillary thyroid carcinoma, knockdown of CCT3 decreased the proliferation and cell cycle progression and induced the apoptosis of K1 cells [11]. In multiple myeloma, CCT3 was also a significant indicator of poor prognosis, and CCT3 expression was associated with the JAK-STAT3 pathway, Hippo signalling pathway, and WNT signalling pathway [12]. In breast cancer, Bassiouni et al. reported that CCT protein level could predict therapeutic application of a cytotoxic peptide [13], and further study shows CCT2 subunit is highly expressed in breast cancer and inversely corelates with patient survival, cells expression CCT2 were more invasive and proliferative. CCT2 depletion prevented tumour growth in a murine model [14].

Genomic analysis of the Cancer Genome Atlas, which contains data for 971 cases of breast carcinoma with sequencing and copy number analysis, showed that 51% of cases have alterations in at least one CCT subunit and that the highest alteration rate occurred in CCT3 (31%) [13]. However, whether CCT3 regulates the development of breast cancer is still unknown.

In the present study, we found that knockdown of CCT3 inhibits the proliferation and metastasis of breast cancer cells and that the mechanism is probably related to regulation of the cell cycle, apoptosis and several signal transduction pathways.

## Materials and methods

### Cells and materials

HCC1937 and MDA-MB-231 cell lines were purchased from the Cell Bank of the Chinese Academy of Science (Shanghai, China); 3-(4,5-dimethylthiazol-2-yl)-2,5-diphenyltetrazolium bromide (MTT) was purchased from Genview (Campbellfield, VIC, Australia); SYBR Master Mixture was purchased from Takara (Shimogyo-ku, Kyoto, Japan); antibodies against CCT3, CDH1, Slug, Snail, VIM, mTOR, ERK1/2, p-ERK1/2, p-AKT1, P38, p-P38, NFκB-65, p-NFκB-65, β-catenin and p-β-catenin were purchased from Cell Signaling Technology (Danvers, MA, USA); and antibodies against CDH2, MMP2, FN1, MYC, p-mTOR and AKT1 were purchased from Abcam (Cambridge, MA, USA). GAPDH antibody was purchased from Santa Cruz Biotechnology, Inc. (Dallas, TX, USA).

### Cell culture

HCC1937 and MDA-MB-231 cell lines were cultured in RPMI 1640 medium (Gibco, Gaithersburg, MD, United States) supplemented with 10% foetal bovine serum (Gibco, Gaithersburg, MD, United States). The cells were maintained at 37 °C in a 5% CO_2_ humidified incubator.

### Lentiviral vector construction and transduction

For the construction of shRNA expression plasmids, shCCT3 was designed based on the target sequence 5′-CAAGTCCATGATCGAAATT-3′. Then, the single strand DNA oligo containing the interference sequence was synthesized, and the double strand DNA was produced by annealing. Then, the two ends of the oligo were directly linked to the lentiviral vector after enzyme digestion. The ligated products were transferred into the prepared Escherichia coli cells. Then, the positive recombinants were identified by PCR and sequenced for verification and plasmid extraction. Lentiviral vector DNA and packaging vectors were transfected into 293T cells. The empty GV115 lentiviral vector was used as the shRNA control (shCtrl). After 48 h of culture, supernatants containing lentiviruses, including shCCT3 and shCtrl, were harvested and purified. Lentiviral transduction was performed on cells at 80% confluency, with a multiplicity of infection (MOI) of 10. Seventy-two hours after infection, the cells were used for downstream assays or transplantation.

### QRT-PCR analysis

Total RNA was isolated by the TRIzol method. The cDNA reverse-transcribed from 250 ng of total RNA was amplified using the following primer sets: CCT3: forward, 5′-TCA GTC GGT GGT CAT CTT TGG-3′, reverse, 5′-CCT CCA GGT ATC TTT TCC ACT CT-3′; and GAPDH: forward, 5′-TGA CTT CAA CAG CGA CAC CCA-3′, reverse, 5′-CAC CCT GTT GCT GTA GCC AAA-3′. Real-time PCR using the SYBR Green PCR Master Mix kit was performed with an ABI Prisma 7000 Sequence Detection System (Applied Biosystems, Foster City, CA, USA) following the manufacturer’s instructions. Data were normalized to the respective GAPDH values. The value of cells infected with shControl(shCtrl) was set to 100% in each run.

### Celigo image cytometry assay

The cells were trypsinized in the logarithmic growth phase, resuspended in medium, seeded into 96-well plates at 2 × 10^3^ cells (100 μl) per well, and incubated overnight. A Celigo Image Cytometer (Nexcelom Bioscience, Lawrence, MA, USA) was used to evaluate the number of cells by scanning for green fluorescence daily for 5 consecutive days at room temperature. The cell proliferation curve was plotted according to the number of cells with green fluorescence.

### MTT assay

HCC1937 and MDA-MB-231 cells infected with shCCT3 or shCtrl were seeded into 96-well plates at 1.5 × 10^3^ cells and 2 × 10^3^ cells per well, respectively, and incubated overnight. The cells were cultured for 5 days at 37 °C. MTT assays were carried out at different time points: 24 h, 48 h, 72 h, 96 h and 120 h. Then, 20 μl MTT solution (5 mg/ml) was added to each well and incubated for an additional 4 h at 37 °C. Then, the MTT solution was aspirated, and 100 μl DMSO was added to dissolve the formazan crystals. The number of cells was counted using a microplate reader at a wavelength of 490 nm.

### Transwell migration assay

HCC1937 and MDA-MB-231 cells infected with shCCT3 or the shCtrl were seeded on Transwell inserts (6.5 mm, 8 μm pores) coated with or without Matrigel in 24-well plates at 60 × 10^3^ cells and 80 × 10^3^ cells per well, respectively, and then placed in the incubator to culture for 24 h. Cells on the upper side of the insert were scraped away, and then the inserts were fixed and stained. Invaded cells were counted under an inverted microscope.

### Flow cytometry analysis

The cells were seeded in 6-well plates for apoptosis analysis or a 6-cm dish for cell cycle analysis. The cells were trypsinized at 70–80% confluency, suspended and washed with D-Hanks solution. For apoptosis analysis, cells were resuspended and stained with annexin V-APC. For cell cycle analysis, cells were fixed with 75% EtOH at − 20 °C for at least 2 h and then harvested and stained with PI (10 ng/ml) and RNase (10 ng/ml). Then, the cells were submitted to flow cytometry analysis.

### Western blot analysis

The proteins were separated by 10% sodium dodecyl sulfate–polyacrylamide gel electrophoresis (SDS-PAGE) (Beyotime, Shanghai, China) and then transferred to polyvinylidene fluoride membranes (Millipore, Billerica, MA, USA), which were blocked for 2 h with 5% nonfat milk. The membranes were incubated with primary antibodies against CCT3 (1:300), CDH1 (1:200), CDH2 (1:1000), Slug (1:1000), MMP2 (1:200), FN1 (1:300), Snail (1:1000), MYC (1:500), VIM (1:1000), ERK1/2 (1:1000), p-ERK1/2 (1:1000), AKT1 (1:1000), p-AKT (1:2000), β-catenin (1:1000), p-β-catenin (1:1000), mTOR (1:1000), p-mTOR (1:2000), NFκB-65 (1:1000), p-NFκB-65 (1:1000), P38 (1:1000), p-P38 (1:1000), and GAPDH (1:2000) overnight at 4 °C. Next, the membranes were incubated with horseradish peroxidase (HRP)-conjugated secondary antibodies (1:2000) for 1 h at room temperature. The blots were visualized using the Super Signal West Femto kit (Pierce Biotechnology, Rockford, IL, USA).

### Rescue experiment

According to the results of signal transduction protein analysis. We selected NFκB-p65 for rescue experiment. MDA-MB-231 cells infected with lentivirus-CCT3-RNAi (Lv-CCT3-RNAi) or lentivirus-NFκB-p65(Lv- NFκB-p65) or lentivirus control were divided into 3 groups: Control group(cells infected with lentivirus control of CCT3-RNAi and NFκB-p65), KD + OE group (Knockdown of CCT3 + Overexpression of NFκB-p65, cells infected with Lv-CCT3-RNAi and Lv-NFκB-p65) and KD + Control group (Cells infected with Lv-CCT3-RNAi and lentivirus control of NFκB-p65). Cell proliferation was tested by celigo image cytometry assay and MTT assay, cell migration was analyzed by trafnswell assay.

### Statistical analysis

All statistical analyses were performed using SPSS 19.0 software (SPSS, Chicago, IL, USA). Data are represented as the mean ± standard deviation. All experiments were performed in triplicate. Student’s t-test or one-way analysis of variance (ANOVA) was used for statistical analysis. For ANOVA, when a significant difference was apparent, Dunnett’s post hoc test was used to compare the means of multiple experimental groups. Differences with p < 0.05 were considered statistically significant.

## Results

### Knockdown of CCT3 expression inhibited breast cancer cell proliferation

**Fig. 1:**
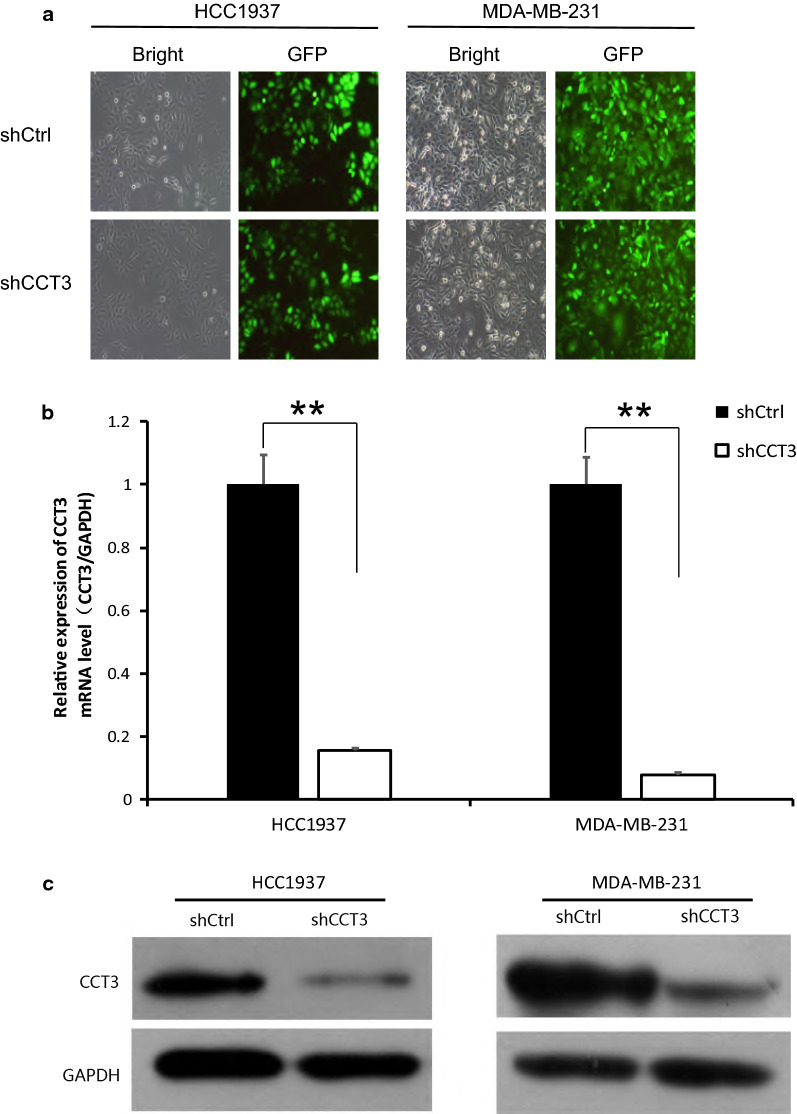
Knock of CCT3 in breast cancer cells. **a** Infection efficiency was determined at 72 h after infection of lentivirus shCCT3 or shCtrl in HCC1937 and MDA-MB-231 cells. Original magnification ×100. **b** CCT3 mRNA in breast cancer cells was measured by realtime RT-PCR, and normalized to GAPDH. Data was presented as the mean ± standard error. **c** CCT3 protein expression was analyzed by western blot analysis in HCC1937 and MDA-MB-231 infected with shCCT3 or shCtrl. **p < 0.01 shCCT3 vs shCtrl

**Fig. 2:**
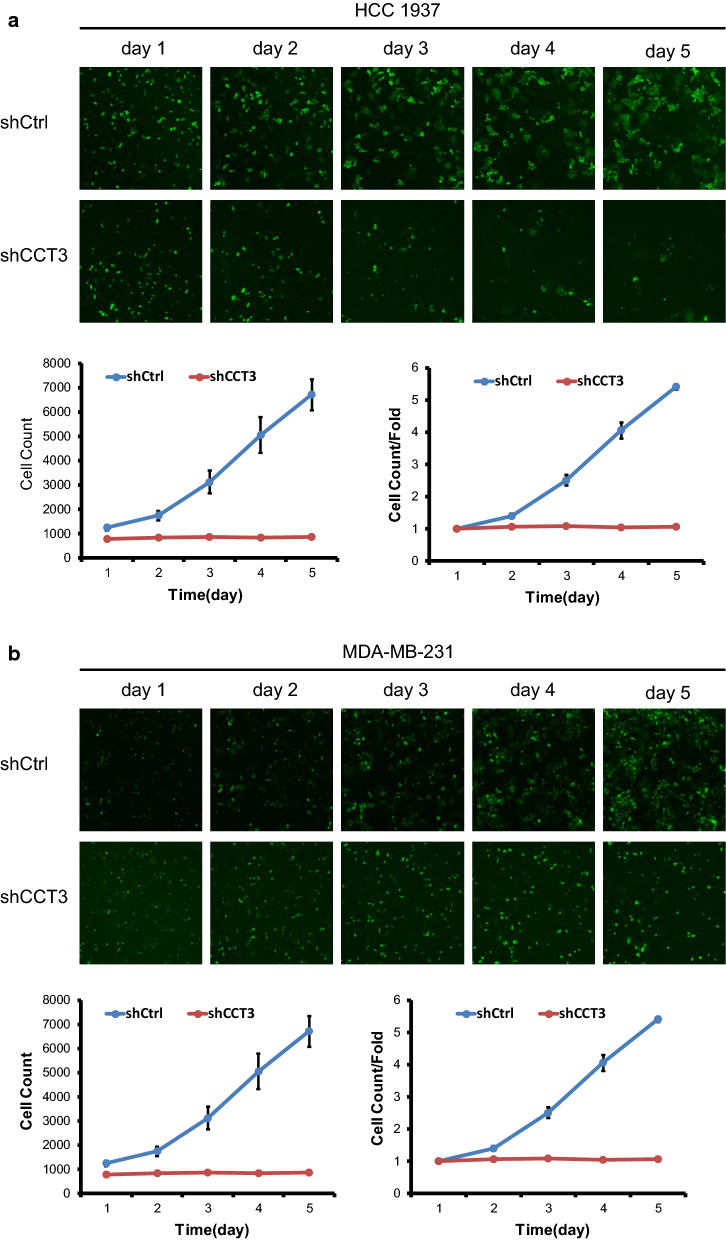
Knockdown of CCT3 suppresses the proliferation of breast cancer cells, HCC1937 (**a**) and MDA-MB-231(**b**) by celigo cytometry analysis. After infection of lentivirus shCCT3 or shCtrl, celigo imgage cytometer was used to evaluate the number of cells by scanning green fluorescence daily for 5 consecutive days and the cell proliferation curve was plotted according to the number of cells with green fluorescence

**Fig. 3:**
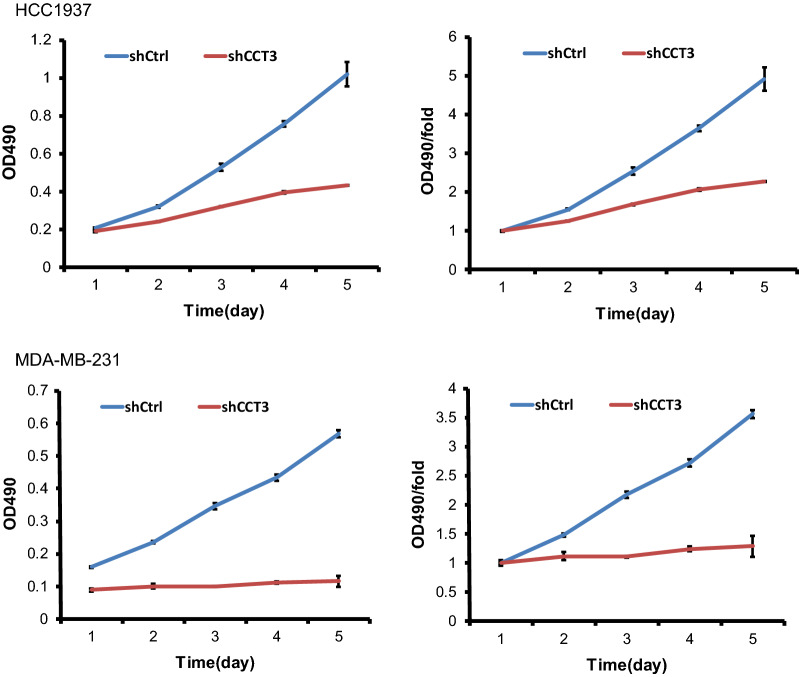
Knockdown of CCT3 suppresses the proliferation of breast cancer cells, HCC1937 and MDA-MB-231 by MTT assay. MTT assay was carried out at 24 h, 48 h, 72 h, 96 h and 120 h after infection of shCCT3 and shCtrl. The number of cells was counted using a microplate reader at a wavelength of 490 nm, and the cell proliferation curve was plotted according to the value of OD490

Lentivirus expressing short hairpin RNA targeting CCT3 mRNA (shCCT3) was used to infect the breast cancer cell lines HCC1937 and MDA-MB-231. As shown in Fig. 1a, the cell transduction rate was above 80%. The silencing effect of CCT3 in HCC1937 and MDA-MB-231 cells was measured by qRT-PCR and western blotting. The expression of CCT3 mRNA decreased by 84.4% and 92.1% after transduction with lentivirus shCCT3 in the HCC1937 and MDA-MB-231 cells compared with that in the shCtrl cells, respectively (Fig. 1b). CCT3 protein expression also decreased after transduction with shCCT3, which was confirmed by western blot analysis (Fig. 1c). Celigo image cytometry assay and MTT assay showed that the proliferation of the breast cancer cells was inhibited significantly in shCCT3 cells compared to shCtrl cells (Figs. 2, 3).

### Knockdown of CCT3 expression inhibited the migration of breast cancer cells

**Fig. 4:**
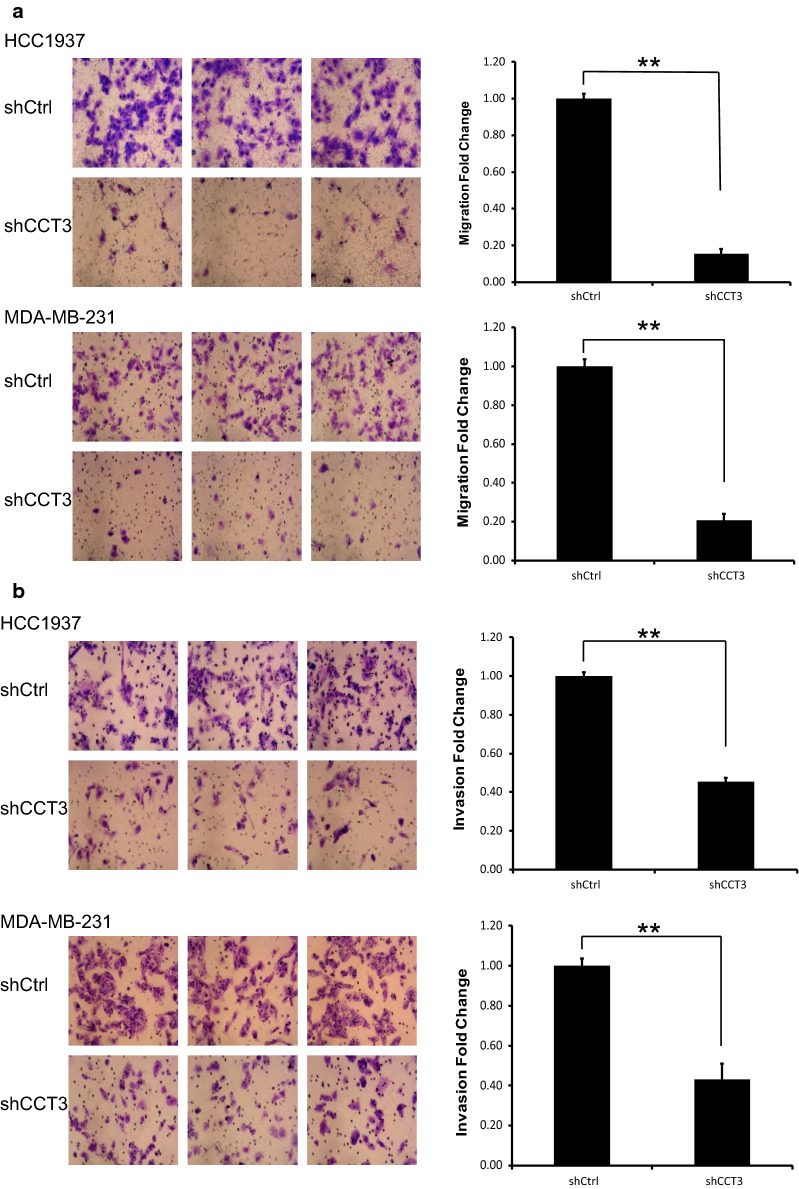
Knockdown of CCT3 promotes the migration of breast cancer cells. **a** Transwell assay without matrigel. **b** Transwell assay with matrigel. The migration of breast cancer cell HCC1937 and MDA-MB-231 was analyzed by transwell assay. Original magnification ×200. Data was presented as the mean ± standard error. **p < 0.01 shCCT3 vs shCtrl

The migration ability of HCC1937 and MDA-MB-231 breast cells was analysed by Transwell assays with or without Matrigel. In Transwell assays without Matrigel, the migration of the shCCT3 group was 16% and 21% of that in the shCtrl group in HCC1937 and MDA-MB-231 cells, respectively. In Transwell assays with Matrigel, the migration in the shCCT3 group was 45% and 43% of that in the shCtrl group in HCC1937 and MDA-MB-231 cells, respectively. As shown in Fig. 4, after transduction with lentivirus shCCT3, the migration of the cells decreased significantly compared to that in the shCtrl group.

### Knockdown of CCT3 promoted apoptosis in breast cancer cells

**Fig. 5:**
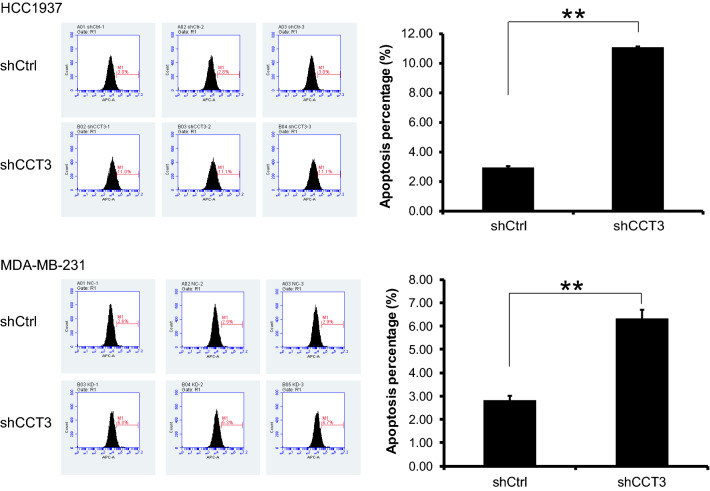
Knockdown of CCT3 induces the apoptosis of breast cancer cells. The apoptosis of breast cancer cell HCC1937 and MDA-MB-231 was analyzed by Annexin V-APC single staining method. Data was presented as the mean ± standard error. **p < 0.01 shCCT3 vs shCtrl

The annexin V-APC single staining method was used for apoptosis analysis. The percentage of apoptotic cells in the shCtrl group was 2.92% and 2.81% and increased to 11.08% and 6.32% in the shCCT3 group in HCC1937 cells and MDA-MB-231 cells, respectively. As shown in Fig. 5, knockdown of CCT3 promoted apoptosis in the breast cancer cell lines HCC1937 and MDA-MB-231.

### Knockdown of CCT3 regulated cell cycle distribution in breast cancer cells

**Fig. 6:**
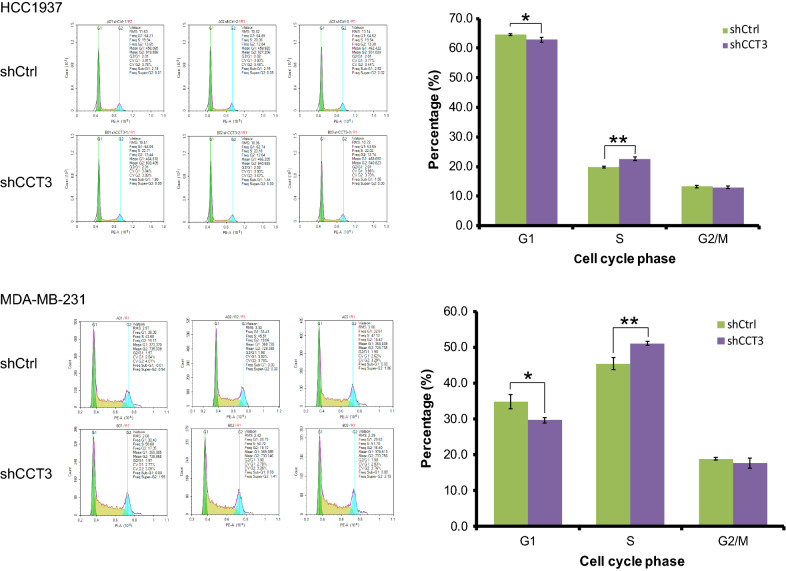
Knockdown of CCT3 regulates the cell cycle process in breast cancer cells. The cell cycle distribution was analyzed by flow cytometry. Data was presented as the mean ± standard error. *p < 0.05, **p < 0.01 shCCT3 vs shCtrl

After transduction with shCtrl or shCCT3 for 5 days, the cell cycle distribution was measured by flow cytometry. The number of cells (HCC1937 and MDA-MB-231) in S phase increased, the number in G1 phase decreased, and there was no significant change in G2/M phase in the shCCT3 group compared with that in the shCtrl group (Fig. 6).

### Knockdown of CCT3 regulated signal transduction proteins in breast cancer cells

**Fig. 7:**
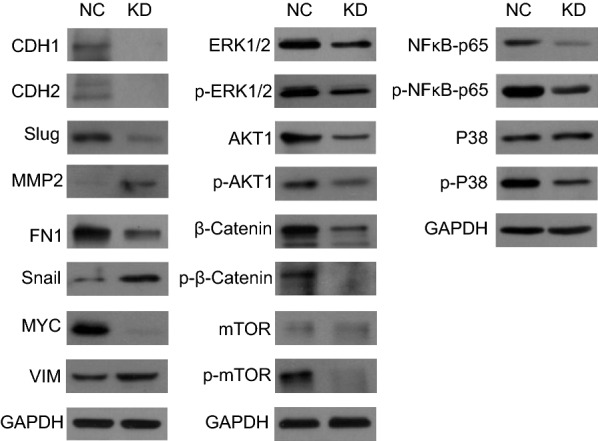
Knockdown of CCT3 regulates signal transduction pathway in MDA-MB-231 cells. The signal transduction protein was measured by western blot analysis, and GAPDH was used as an inner control

To explore possible signal transduction pathways affected by the CCT3 gene, we detected several signal transduction proteins by western blot analysis in MDA-MB-231 cells. As shown in Fig. 7, the results showed that MMP2, Snail, and VIM were upregulated and that CDH1, CDH2, Slug, FN1, MYC, ERK1/2, p-ERK1/2, AKT1, p-AKT1, β-catenin, p-β-catenin, p-mTOR, NFκB-p65, p- NFκB-p65 and p-P38 were downregulated in MDA-MB-231 cells infected with shCCT3 compared to cells infected with shCtrl. There were no significant differences in mTOR and P38 between the 2 groups.

### Overexpression of NFκB-p65 rescued the cell proliferation and migration affected by CCT3 in breast cancer cells

**Fig. 8:**
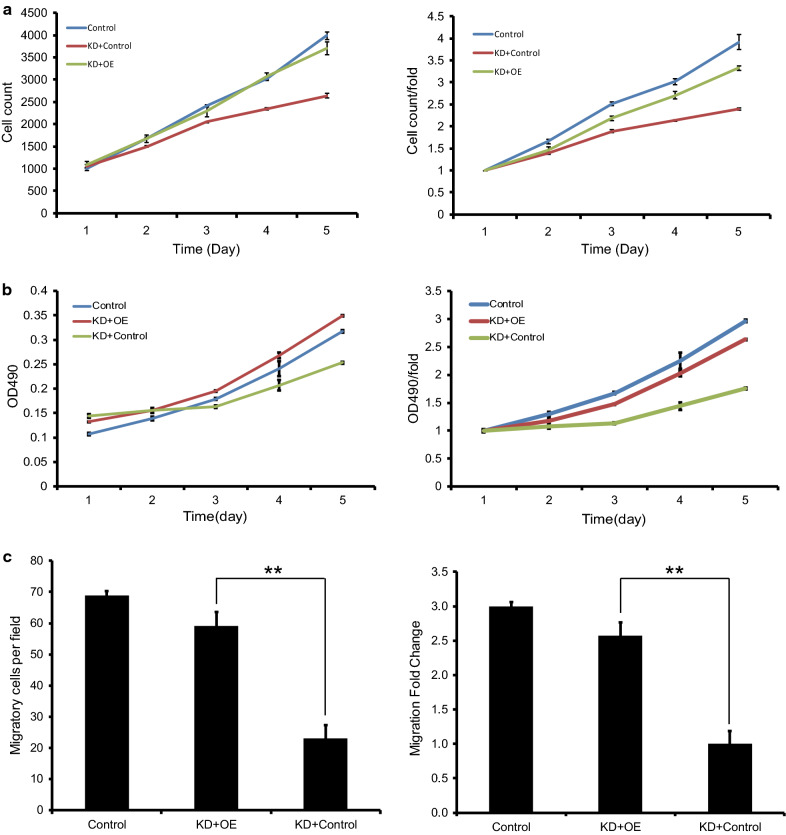
Overexpression of NFκB-p65 rescued the cell proliferation and migration affected by CCT3 in breast cancer cells. Celigo image cytometry assay (**a**) and MTT assay (**b**) showed that the proliferation of the breast cancer cells was inhibited significantly in KD + Control cells compared to control cells, and overexpression of NFκB-p65 (KD + OE) rescued the effect of CCT3. Transwell migration assay showed that the migration of breast cells was also rescued by overexpression of NFκB-p65 (**c**). Control group: cells infected with lentivirus control of CCT3-RNAi and NF-κB-p65; KD + OE: Knockdown of CCT3 + Overexpression of NF-κB-p65, cells infected with CCT3-RNAi and lentivirus-NF-κB-p65; KD + Control: Cells infected with CCT3-RNAi and lentivirus control of NF-κB-p65

To explore the roles of NFκB-p65 signaling pathways in cell proliferation and migration affected by CCT3 in breast cancer cells (MDA-MB-231), a rescue experiment was carried out. Celigo image cytometry assay (Fig. 8a) and MTT assay (Fig. 8b) showed that the proliferation of the breast cancer cells was inhibited significantly in KD + Control cells compared to control cells, and overexpression of NFκB-p65 in KD cells (KD + OE group) rescued the effect of CCT3. Transwell migration assay showed that the migration of breast cells was also rescued by overexpression of NFκB-p65(Fig. 8c).

## Discussion

Currently, the treatments for breast cancer include surgery, endocrine therapy, radiation therapy, chemotherapy, and targeted therapy. Among them, targeted therapy, such as trastuzumab, CDK4/6 inhibitors, and PI3K/Akt/mTOR inhibitors, significantly prolongs the survival time of patients with breast cancer [15–17]. However, the overall survival is not satisfactory, especially in patients with advanced breast cancer. Therefore, we wanted to search for a novel therapeutic target to improve the therapeutic effect and prolong the survival time.

CCT3 is an important subunit of the molecular chaperone CCT and is involved in the folding process of 7% of all cytosolic proteins, such as cytoskeletal proteins (tubulins, actins), cyclin E and Von Hippel-Lindau (VHL) [6, 18, 19]. These proteins determine the central role of CCT in the proliferation of cancer cells. The expression of CCT3 in cancer tissue is higher than that in non-cancerous tissue, and knockdown or suppression of the expression of CCT3 can inhibit the proliferation of cancer cells in many malignant carcinomas, such as hepatocellular carcinoma [9], gastric carcinoma [10], and papillary thyroid carcinoma [11]. In our study, we found that transduction with the lentiviral shRNA targeting CCT3 suppressed the mRNA and protein expression of CCT3 in the breast cancer cell lines HCC1937 and MDA-MB-231. MTT assay and Celigo analysis showed that knockdown of CCT3 inhibited the proliferation of breast cancer cells, which is consistent with reports in other tumours.

Rearrangement of actin filaments plays an important role in cancer cell migration or invasion [20]. P-21-activating kinase PAK4 and gelsolin are actin regulators that are known to bind to CCT [21, 22]. Therefore, inhibiting the expression of CCT should decrease the migration ability of cancer cells. Indeed, many reports have shown that knockdown of CCT inhibits the migration and invasion of some cancer cells [23–25]. In our study, we also found that knockdown of CCT3 inhibited the migration of breast cancer cells through Transwell analysis.

The relationship between CCT and the cell cycle has been well reported [6, 26]. Many cell cycle regulatory proteins are substrates of CCT, such as tubulin, Cdc20, and Cdh1. Tubulin synthesis increases around the G1/S transition, and a CCT-tubulin interaction has been observed in early S phase [27]. Cdc20 and Cdh1 are important at the transition from metaphase to anaphase [28]. Therefore, many reports have shown that suppression of CCT3 can induce S phase arrest. In this study, we found that knockdown of CCT3 increased the number of cells in S phase and decreased the number of cells in G1 phase, while the number of cells in G2/M phase was not significantly altered.

Apoptosis plays an important role in the carcinogenesis, development and treatment of breast cancer [29]. It has been reported that inhibition of CCT3 can induce apoptosis [11]. We confirmed that knockdown of CCT3 can induce apoptosis in breast cancer with the annexin method in this study. Perhaps the mechanism is related to Cdc20 and p53. As mentioned above, Cdc20 and p53 are substrates of CCT. Cdc20 is known to modulate key anti-apoptic proteins Mcl-1 and Bim [30, 31], and p53 mediates cell apoptosis by activating mitochondrial pathway and death receptor-induced apoptotic pathway [32].

CCT3 is involved in STAT3 protein folding [33], and many reports have confirmed that the effect of CCT3 is achieved via the JAK-STAT3 pathway. Therefore, we tried to explore other signalling pathways found that CCT3 can regulate a variety of signalling pathways in breast cancer cells, some of which play an important role in tumour development. The results showed that Snail, VIM and MMP2 were upregulated and that CDH1, CDH2, ERK1/2, p-ERK1/2, p-P38, FN1, AKT1, p-AKT1, MYC, NFκB-p65, p- NFκB-p65, p-mTOR, β-catenin, p-β-catenin and Slug were downregulated in MDA-MB-231 cells infected with shCCT3 compared to those infected with shCtrl. Many reports showed that NFκB plays an important role in the proliferation and migration in breast cancer [34–36], therefore we selected it for rescue experiment. The results showed that overexpression of NFκB rescued the effect of CCT3 on the proliferation and migration of breast cancer cells. In future studies, rescue experiments of other signal transduction protein and mechanism studies will be carried out to confirm the role of these signal transduction pathways in breast cancer and to explore the mechanism between CCT3 and signal transduction pathways.

## Conclusions

In our study, we found that knockdown of CCT3 can inhibit the proliferation and metastasis of breast cancer cells, induce apoptosis and regulate the cell cycle. The effect of CCT3 may be achieved through a variety of signalling pathways. CCT3 may be a novel therapeutic target for breast cancer.

## Data Availability

All data generated or analysed during this study are included in this published article.

## Abbreviations

HSP 60: Heat shock protein 60
CCT: Chaperonin-containing TCP-1
Cell division cycle: cdc
KRAS: Kirsten rat sarcoma viral oncogene
STAT3: Signal transducers and activators of transcription 3
HCC: Hepatocellular carcinoma
MTT: 3-(4,5-Dimethylthiazol-2-yl)-2,5-diphenyltetrazolium bromide
MOI: Multiplicity of infection
SDS-PAGE: Sodium dodecyl sulfate–polyacrylamide gel electrophoresis
KD: Knockdown
OE: Overexpression
ANOVA: One-way analysis of variance
VHL: Von Hippel-Lindau
